# Ambulatory Assessment in Concussion Clinical Care and Rehabilitation

**DOI:** 10.3389/fdgth.2022.924965

**Published:** 2022-06-23

**Authors:** R. J. Elbin, Melissa N. Womble, Daniel B. Elbich, Christina Dollar, Sheri Fedor, Jonathan G. Hakun

**Affiliations:** ^1^Department of Health, Human Performance and Recreation/Office for Sport Concussion Research, University of Arkansas, Fayetteville, AR, United States; ^2^Inova Sports Medicine Concussion Program, Fairfax, VA, United States; ^3^Department of Neurology, College of Medicine, The Pennsylvania State University, Hershey, PA, United States; ^4^Department of Psychology, The Pennsylvania State University, State College, PA, United States; ^5^Center for Healthy Aging, The Pennsylvania State University, State College, PA, United States; ^6^Translational Brain Research Center, College of Medicine, The Pennsylvania State University, Hershey, PA, United States

**Keywords:** concussion, ambulatory cognitive assessment, EMA, rehabilitation concussion ambulatory assessment, rehabilitation, exposures

## Abstract

Concussion is a mild traumatic brain injury that is characterized by a wide range of physical, emotional, and cognitive symptoms as well as neurocognitive, vestibular, and ocular impairments that can negatively affect daily functioning and quality of life. Clinical consensus statements recommend a targeted, clinical profile-based approach for management and treatment. This approach requires that clinicians utilize information obtained via a clinical interview and a multi-domain assessment battery to identify clinical profile(s) (e.g., vestibular, mood/anxiety, ocular, migraine, cognitive fatigue) and prescribe a corresponding treatment/rehabilitation program. Despite this comprehensive approach, the clinical picture can be limited by the accuracy and specificity of patient reports (which often conflate timing and severity of symptomology), as well as frequency and duration of exposure to symptom exacerbating environments (e.g., busy hallways, sitting in the back seat of a car). Given that modern rehabilitation programs leverage the natural environment as a tool to promote recovery (e.g., expose-recover approach), accurate characterization of the patient clinical profile is essential to improving recovery outcomes. Ambulatory assessment methodology could greatly benefit concussion clinical care by providing a window into the symptoms and impairments experienced by patients over the course of their daily lives. Moreover, by evaluating the timing, onset, and severity of symptoms and impairments in response to changes in a patient's natural environment, ambulatory assessments can provide clinicians with a tool to confirm clinical profiles and gauge effectiveness of the rehabilitation program. In this perspective report, we review the motivations for utilizing ambulatory assessment methodology in concussion clinical care and report on data from a pilot project utilizing smart phone-based, ambulatory assessments to capture patient reports of symptom severity, environmental exposures, and performance-based assessments of cognition for 7 days following their initial evaluation.

## Introduction

Concussion, or mild traumatic brain injury (mTBI), is characterized by a heterogenous presentation of signs, symptoms, and impairments that can negatively affect physical, emotional, cognitive, and sleep-related functioning. A clinical care approach that includes a multi-domain assessment to inform an active, targeted treatment plan is key to mitigating poor recovery outcomes. Concussion recovery time is variable, with most patients recovering within 30 days of injury and a smaller percentage of patients exhibiting a longer, protracted recovery (1–4). Several patient- (e.g., concussion history) and injury-related risk factors (e.g., on-field dizziness) are associated with protracted recovery and warrant consideration by the clinician (5, 6). The current standard of care for concussion includes a patient interview (e.g., health history, injury details) and a comprehensive battery of symptom, neurocognitive, vestibular, ocular, and mood assessments (7, 8). Recent clinical and expert consensus (7, 9–11) advocate for a clinical profiles-based care approach that utilizes subjective reports from patients and objective, multi-domain assessment data to identify a concussion clinical profile that can be matched to a targeted treatment and rehabilitation strategy [e.g., a vestibular profile would warrant referral to vestibular therapy; (12, 13)]. The timeline for concussion patient care includes an initial clinical visit (ideally within the first week of injury) where a concussion clinical profile(s) is determined, and a treatment plan is established. Follow-up visits to the clinic occur ~every 1–2 weeks where the clinician will gauge progress and recovery until medical clearance.

The centerpiece of the concussion clinical evaluation involves ascertaining the patient's lived injury experience. The accuracy of this information is critical for the clinician to generate a clear picture of the injury and establish a targeted treatment plan (8, 14). Self-reported symptoms are the primary driver of clinical care, and these reports are associated with determining injury severity, clinical profile and treatment, and risk for protracted recovery (3, 15–18). Beyond the diversity and severity of reported symptoms, the impact of patients' everyday life environments on these symptoms is also very informative to the clinician. For example, dizziness may be a patient's chief complaint; however, the clinician may inquire how different daily environments and activities affect dizziness (e.g., riding in a car, shopping at a store). The value of this information has prompted researchers to modify current symptom scales to be more inclusive of the interaction between the patient's environment and their symptoms (e.g., the Clinical Profiles Screen: CP Screen) (19).

While patient reports are essential to constructing the clinical picture, encapsulation of this information within the clinical interview has its limitations. Patient reported outcomes may lack ecological validity, as they may be influenced by a number of patient attributes [e.g., somatization (20, 21), sport ethic (22), parental influence (23)], and performance-based assessments are obtained under controlled testing conditions (e.g., quiet, clinical testing rooms often with parents or caregivers present). Moreover, patients may struggle to provide accurate, retrospective accounts of the type, frequency, and severity of their concussion symptoms over the course of several clinic visits due to recall bias, lack of education, awareness, and short-lived experience with their concussion. These limitations often require clinicians to estimate the burden of symptoms and impairments in their patients, which may influence treatment selection and recovery outcomes.

## Ambulatory Assessment in Acute Concussion Management

Our approach intends to improve assessment and monitoring by conducting short surveys and ultra-brief cognitive assessments (typically <60 s) on smart phones in a measurement burst design (24–27). By delivering these assessments remotely, and over the course of participants' daily lives, we hope to capture a more accurate representation of patients' everyday experiences including the frequency and duration of exposures to various, potentially symptom-modifying, environments (28, 29). By assessing symptom severity in close temporal proximity to experience, we aim to reduce the influence of biases associated with patient reported outcomes [e.g., retrospective biases (29, 30)]. An advantage of conducting ambulatory cognitive assessments in such an intensive, longitudinal study design protocol is the ability to distribute an equivalent or greater amount of overall testing that would be completed in the clinic, over the course of a week between visits. Separating performance-based assessments from the clinical environment allows patients to complete the assessments in more familiar surroundings (e.g., home or school), increasing the ecological validity of the testing context (24, 27). For example, some patients exhibit significant anxiety related to completing neurocognitive testing for their concussion and discussing their symptoms with the clinician. The administration of performance-based assessments in the patient's daily life and environments may not only reduce anxiety, but possibly increase the clinical accuracy of these assessments and self-reported data from patients. In addition, intensive repeated assessment may help reveal the timecourse of recovery by generating a semi-continuous timeseries of symptom and cognitive performance data.

## The Mobile Neurocognitive Health Project

The Mobile Neurocognitive Health project is a study of middle and high school athletes with sport-related concussion. Participants were recruited based on the following inclusion criteria: 13+ years of age, medically diagnosed concussion occurring within 7 days of enrollment, no history of a prior concussion within the last 6 months, and willing to use personal smart phone for data collection. Participants were excluded if there was evidence of more severe brain injury (e.g., positive neuroimaging finding, loss of consciousness ≥20 min), they were not fluent in English, or had significant visual impairments that would prevent use of a smart phone. International consensus criteria for concussion were used to standardize injury diagnosis for all participants (7). All participants were enrolled in the study protocol during acute injury presentation (<7 days of injury). Participants in MNCH are asked to download our study application (the Mobile Monitoring of Cognitive Change, *M2C2*, smart phone application, under development as part of the forthcoming, NIH *Mobile* Toolbox) onto their own phones and respond to 4 assessment notifications daily, for a period of 7 days following their initial clinic visit. Two feasibility questions addressed by MNCH include: (a) whether students with a sport-related concussion would comply with a measurement burst design protocol, and (b) whether cognitive exertion associated with completing the surveys and ultra-brief cognitive assessments might exacerbate symptoms.

**Figure 1:**
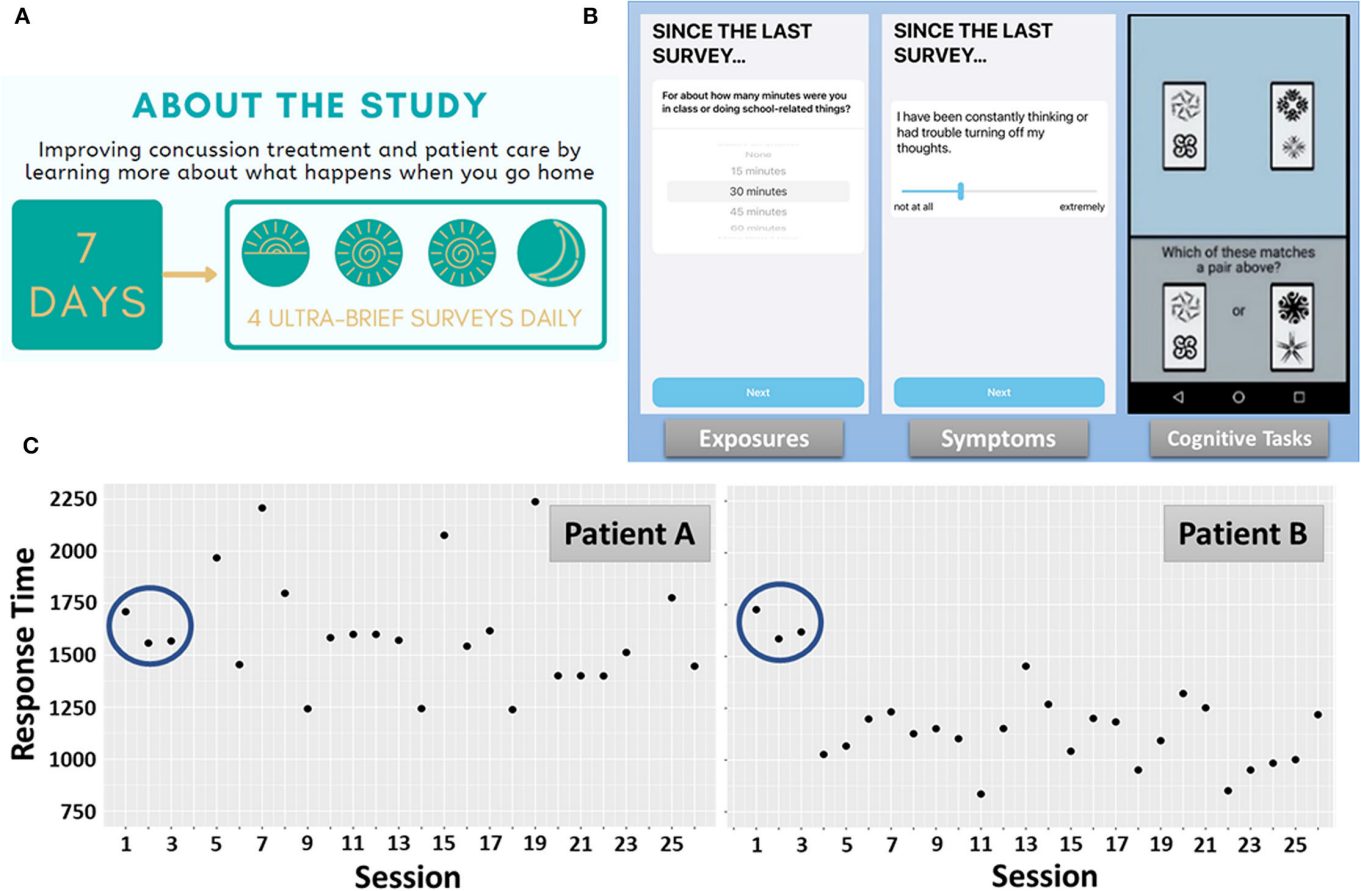
MNCH Study Protocol. **(A)** Recruitment study infographic depicting the measurement burst design. **(B)** Each assessment contained short surveys of various environmental exposures, symptom severity ratings, and performance-based cognitive tasks. The Symbol Search task, measuring processing speed and attention, is depicted. **(C)** Response time profiles during Symbol Search performance over the 7-day measurement burst. Patient A and Patient B exhibit nearly identical response times during the first 3 administrations of the Symbol Search task. Thereafter, Patient A exhibits high intra-individual variation and generally no improvement in task performance over 7 days. Patient B, by comparison, exhibits exponential improvement early in the measurement burst, lower intra-individual variation, and evidence of incremental improvements throughout the measurement burst.

Conducting a measurement burst design study involving student athletes requires tailoring of the assessment protocol to the demands of student life, while also providing space for day to day variation in schedule and availability (e.g., weekday vs. weekend). Accordingly, assessments are scheduled during four 2.5-h time windows throughout each day (approximating the start of the school day, lunch, the end of school day, and evening prior to bedtime; Figure 1A). Data from the first 60 participants suggest that 75% of patients provided evaluable data (completing >50% expected assessments), including a 78.6% median compliance rate to the ambulatory protocol (equivalent to completing ~22 of the 28 assessments administered). Initial results from a single item administered at the end of each assessment (“*Completing the assessments made my symptoms worse.”* 100-pt visual analog slider: *Not at All* to *Extremely*) suggest that the MNCH ambulatory assessments are generally well-tolerated (*M* = 34 pts out of 100, *SD* = 24.6). Overall, our initial observation is that ambulatory assessment of exposures, symptoms, and cognition in a measurement burst design may be acceptable in this population, among which compliance and exertion are important considerations.

## Performance-Based Ambulatory Cognitive Assessments

In-person computerized or paper-and-pencil neuropsychological assessment represents the current standard for performance-based assessment of cognition during concussion management and rehabilitation (7). However, periodic, clinic-based assessments provide a relatively low resolution timecourse of neurocognitive recovery. Distributed ultra-brief cognitive assessments can provide a timecourse of data describing change over multiple timescales (moment-to-moment, day-to-day, over the course of a week). Multiple features of this timecourse may provide clinically-meaningful information about the recovery trajectory.

Figure 1B depicts one trial of the M2C2 Symbol Search task and Figure 1C depicts median response times (RT) for each administration (“session”), over the 7-day measurement burst, for 2 participants. While Patient A and Patient B exhibit nearly identical performance during the first 3 sessions, highly differentiable patterns of performance unfold over the remainder of the measurement burst. Of note, Patient A exhibits greater session-to-session, intra-individual variation than Patient B. Inconsistency in speeded performance in other populations (e.g., older adults) has been identified as a risk factor for cognitive impairment (31–33). Recently, in a study using M2C2, we found that older adults with mild cognitive impairment exhibit significantly greater session-to-session and day-to-day variation in Symbol Search task performance than cognitively-unimpaired older adults (34). A primary aim of the MNCH project is to determine if RT inconsistency is a clinically-relevant marker of cognitive impairment following concussion.

A second feature that differentiates Patient A from Patient B is the apparent lack of improvement in performance over the 7-day burst. Retest effects are common among performance-based assessments and may reflect procedural learning (e.g., familiarity with timing and task procedures and adaptation to response modality) or explicit learning of testing materials (35–38). M2C2 assessments such as the Symbol Search task are designed to discourage explicit learning as stimuli are randomly drawn from large stimulus banks on each trial. Nonetheless, we have observed substantial retest effects during Symbol Search task performance that are well-captured by process models of learning and proceduralization [negative exponential modeling (39, 40)]. An important consideration for monitoring of concussion is that improvements are likely to reflect the joint contribution of retest learning and recovery from the injury. That is, concussion recovery is associated with improvements in processing speed (2, 41–45), and retest effects should trend in the same direction. Application of cognitive process models may help dissociate retest- from recovery-related improvements. Notwithstanding, an overall lack of improvement in RT (as seen with Patient A) is reported to be a predictive marker for protracted recovery [see *Future Directions* (42, 44)].

## Everyday Exposures and Symptom Severity Ratings

Exposure to potentially symptom-modifying environments and activities is cataloged during each assessment in MNCH. We surveil 9 environments and activities that student athletes are commonly exposed to during an average school day and weekend (19). During each assessment participants are asked to report the approximate amount of time they spent in each of these environments and activities. Figure 1B displays an example of an exposure assessment related to school activities. From this data stream we can extrapolate both the frequency of exposure to each environment and activity, as well as the average duration, over the course of the 7-day burst period. Following exposure and activity reporting, participants in MNCH are asked to rate the severity of 15 commonly reported concussion symptoms (46). Symptom severity ratings are made on a 100 pt visual analog scale using a slider element. Figure 1B displays an example symptom severity rating of perseverative thought.

**Figure 2:**
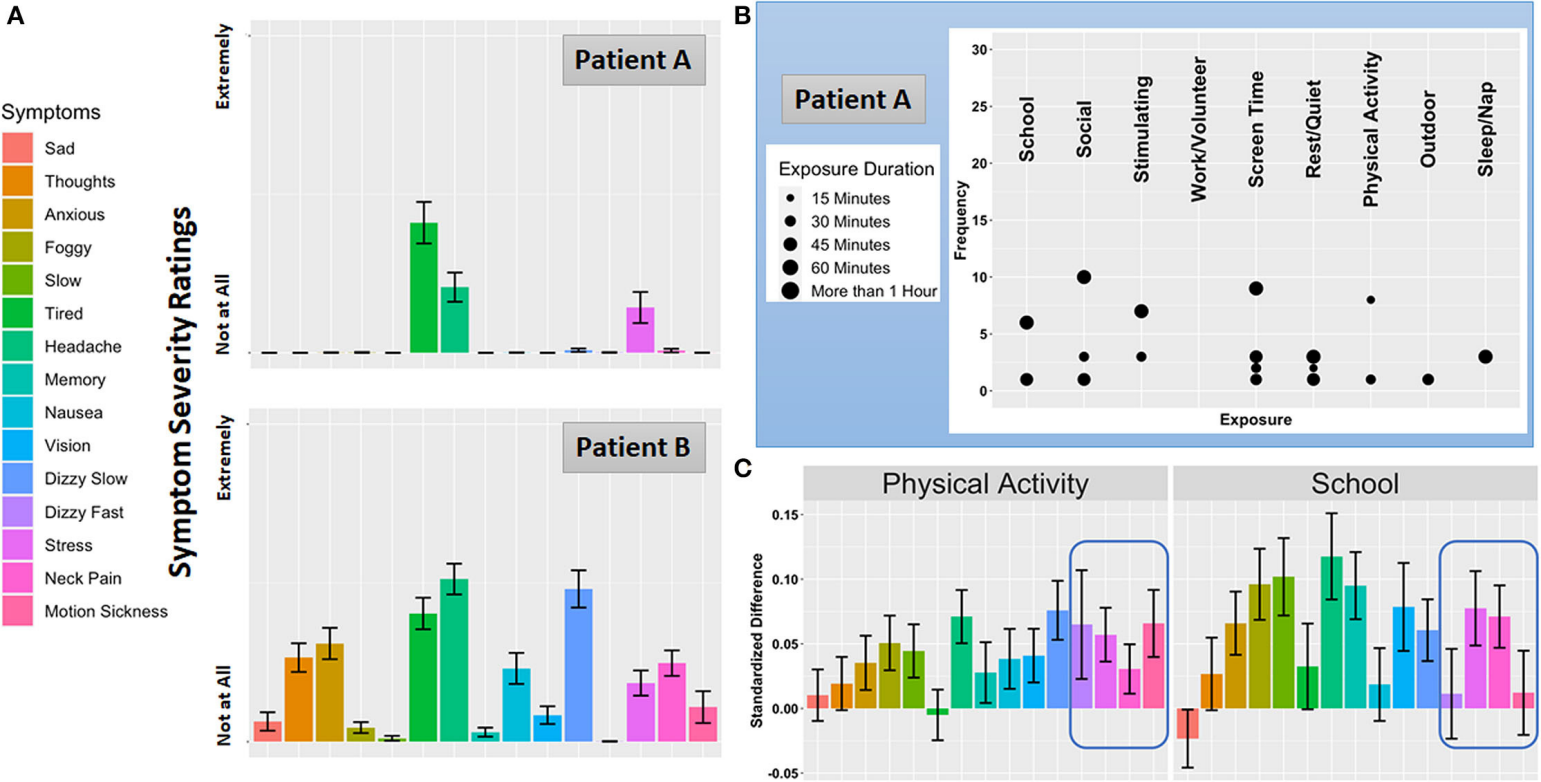
Symptom Severity, Exposure and Activity Reports, and Context-dependent Symptom Severity Change. **(A)** Average symptom ratings for Patient A and Patient B over the burst. **(B)** Frequency and duration of the 9 everyday environments and activities Patient A reported as exposures. Consistent with the daily life a student athlete, Patient B's exposure pattern was very similar to Patient A and not displayed here. **(C)** Exposure-specific symptom reactivity to two common everyday exposures for participants enrolled in the MNCH project.

Figure 2A displays average symptom severity ratings for the same two patients depicted in Figure 1. Patient A reports a cluster of symptoms centered around feelings of tiredness, headache, and stress, which are thought to be indicative of a primary post-traumatic migraine profile (3, 47). In contrast, while Patient B reports an overlapping pattern of tiredness, headache, and stress, they also report experiencing symptoms consistent with an anxiety/mood profile (perseverative thoughts, anxiety) as well as vestibular impairments (experience of slow dizziness and nausea). These characteristics of Patient B are consistent with the frequent and overlapping of the anxiety and vestibular clinical profiles of concussion (12). An important aim of MNCH will be to compare ambulatory symptom severity ratings with results of clinical interviews to potentially validate putative injury profiles. Moreover, modeling the degree to which symptoms co-occur, vary over time, and/or operate in a lead-lag fashion over daily life will help highlight symptoms or symptom clusters that might be viable targets for precision concussion rehabilitation (see *Future Directions*). Finally, similar to data derived from the ambulatory cognitive assessments, symptom timecourses may provide an invaluable monitoring system for tracking recovery as well as the efficacy of rehabilitation programs.

## Reactivity to Everyday Exposures

Although the influence of everyday exposures on concussion symptoms and impairments is central to the clinical concussion evaluation, empirical reports on this topic are scant. To date researchers have focused primarily on the exposure to academic (48, 49) and physical activity (50–52) environments. Natural exposure to the academic environment (i.e., attending a day of school with no accommodations for concussion) is linked to increased symptoms and prolonged recovery (48, 53), which has prompted the adoption of a gradual “return to learn” care plan or “pacing” model (54, 55). The usage and frequency of accommodations have been described, but this literature is limited by cross-sectional, cohort studies. Aerobic-based, expose-recover approaches (50, 56) that focus on sub-symptom physical activity is linked to improved concussion outcomes, but these exposures occur during bouts of supervised exercise, not measured in the patient's everyday life. Critically, it remains poorly understood whether the same exposures consistently modify the same symptoms across patients in their natural environment. Precision rehabilitation will require an understanding of which environments and activities affect which patients in which ways. Figure 2C shows how two frequent everyday exposures influence each symptom monitored in MNCH. Standardized differences in symptom severity ratings are plotted for moments where patients report being exposed to physical activity and school activity compared with moments where these exposures are not reported. While physical activity and school activities are associated with increases in overlapping symptoms, the highlighted region shows how feelings of fast dizziness and motion sickness are specific to physical activity, which is consistent with clinical anecdotes suggesting that clusters of symptoms may be closely tied to specific everyday exposures.

## Future Directions

Ambulatory assessments represent a new tool for both basic science and clinical management of concussion. As is highlighted by individual data from the MNCH study, ambulatory assessments can be leveraged to provide complementary information to in-clinic evaluation and generate real-time feedback on symptom response to everyday environmental and activity exposures. This information could be used to corroborate the clinical impression (e.g., confirm clinical profile) and to monitor the efficacy of treatment plans. A key contribution may also be the use of ambulatory assessment to optimize, and gauge adherence to, post-concussion management recommendations and rehabilitation prescriptions. For example, behavioral management recommendations are often used as a first-step management approach for concussion and includes proper sleep hygiene, nutrition, hydration, physical activity, and stress management (12). Post-concussion rehabilitation strategies may include vestibular and/or ocular motor therapy referral, which involve both in-clinic and at-home therapy sessions that involve exposure to symptom-provoking environments in a controlled manner [i.e., expose-recover (57)]. Higher density data streams could be leveraged to document adherence and effectiveness of behavioral regulation strategies and determine optimum dosage (i.e., duration) of expose-recover rehabilitation strategies.

Ambulatory assessments may also be leveraged as a digital biomarker of injury severity and recovery. Neurocognitive impairments are one concussion hallmark, and a digital biomarker of concussion injury and recovery will require sensitivity to change over multiple timescales. Our approach is designed to maximize sensitivity to several aspects of cognitive change including changes in level and variation in performance. We anticipate that short-term dynamics (absence of short-term improvement/high intra-individual variation) might be a valuable predictive digital biomarker of protracted recovery. Despite exhibiting overall greater symptom burden and severity than Patient A, Patient B exhibited a pattern of performance on the Symbol Search task consistent with retest learning (exponential improvements) and lower moment-to-moment variation over the measurement burst (Patient A exhibited no overall improvements). Although both patients, similar in age (15 and 17 years) and both female, began the measurement burst within 4 days of their injury; Patient B was medically cleared after 10 days compared to 54 days for Patient A. A primary aim of the MNCH project is to determine whether these short-term neurocognitive outcomes predict protracted recovery and document how these outcomes might be associated with or modified by other established risk factors (e.g., symptom burden). In addition, we aim to establish the utility of performance-based ambulatory cognitive assessments as a digital biomarker of recovery. This effort will require establishing norms and expected range of performance (and variation) in healthy individuals as well as patients during the course of the injury. Against this backdrop, the efficacy of novel rehabilitation programs could be tested and evaluated at multiple timescales (i.e., acute vs. longer-term outcomes). Finally, performance-based, ambulatory cognitive assessments offer an opportunity to confirm the temporality of impairments (e.g., evidence of impairments after episodes of cognitive exertion/school in patients with a cognitive/fatigue profile) and disruption of regulatory systems (e.g., changes in diurnal patterns).

The remote nature of ambulatory assessments creates new avenues both early in the injury and into longer-term recovery. Health care disparities due to geographical or economic circumstances could be reduced by delivering these assessments and rehabilitation prescriptions over pervasive technology (i.e., smart phones), reaching individuals without access to specialty care. In addition, ambulatory assessment protocols that require limited proctoring or onboarding could be a tool for athletic trainers to deploy in regular practice, which may help refine the referral process to primary or specialty care (i.e., support triage based on remote screening). Finally, the ability to conduct longer-term, remote monitoring (i.e., telemedicine) might help better define the bounds of medical clearance/clinical recovery as ambulatory assessments offer a low-burden, remote approach to repeated assessment during follow-up.

## Data Availability Statement

The raw data supporting the conclusions of this article will be made available by the authors, without undue reservation.

## Ethics Statement

The studies involving human participants were reviewed and approved by WCG IRB. Written informed consent to participate in this study was provided by the participants' legal guardian/next of kin.

## Author Contributions

JH, RE, and MW contributed to the conception of the study design. CD coordinated the study and conducted all data collection. DE conducted all data processing and analyses. JH and RE wrote the first draft of the manuscript. MW and SF wrote sections of the manuscript. All authors contributed to manuscript revision, editing, and approved the submitted version.

## Funding

This study was supported by the National Institute on Aging of the National Institutes of Health under award numbers R00AG056670 and U2CAG060408. JH's effort on the project was supported by R00AG056670. Funding for the study costs was supported by the Penn State College of Medicine through research startup funding provided to JH. Support for development and instrumentation of the ambulatory assessment platform was provided by U2CAG060408. The content is solely the responsibility of the authors and does not necessarily represent the official views of these granting agencies.

## Conflict of Interest

The authors declare that the research was conducted in the absence of any commercial or financial relationships that could be construed as a potential conflict of interest.

## Publisher's Note

All claims expressed in this article are solely those of the authors and do not necessarily represent those of their affiliated organizations, or those of the publisher, the editors and the reviewers. Any product that may be evaluated in this article, or claim that may be made by its manufacturer, is not guaranteed or endorsed by the publisher.
